# The role of VosA/VelB-activated developmental gene *vadA* in *Aspergillus nidulans*

**DOI:** 10.1371/journal.pone.0177099

**Published:** 2017-05-08

**Authors:** Hee-Soo Park, Mi-Kyung Lee, Sun Chang Kim, Jae-Hyuk Yu

**Affiliations:** 1 School of Food Science and Biotechnology, Institute of Agricultural Science and Technology, Kyungpook National University, Daegu, Republic of Korea; 2 Departments of Bacteriology and Genetics, University of Wisconsin, Madison, WI, United States of America; 3 Department of Biological Sciences, Korea Advanced Institute of Science and Technology, Daejeon, Republic of Korea; Soonchunhyang University, REPUBLIC OF KOREA

## Abstract

The filamentous fungus *Aspergillus nidulans* primarily reproduces by forming asexual spores called conidia, the integrity of which is governed by the NF-κB type *velvet* regulators VosA and VelB. The VosA-VelB hetero-complex regulates the expression of spore-specific structural and regulatory genes during conidiogenesis. Here, we characterize one of the **V**osA/VelB-**a**ctivated **d**evelopmental genes, called *vadA*, the expression of which in conidia requires activity of both VosA and VelB. VadA (AN5709) is predicted to be a 532-amino acid length fungal-specific protein with a highly conserved domain of unknown function (DUF) at the N-terminus. This DUF was found to be conserved in many Ascomycota and some Glomeromycota species, suggesting a potential evolutionarily conserved function of this domain in fungi. Deletion studies of *vadA* indicate that VadA is required for proper downregulation of *brlA*, *fksA*, and *rodA*, and for proper expression of *tpsA* and *orlA* during sporogenesis. Moreover, *vadA* null mutant conidia exhibit decreased trehalose content, but increased β(1,3)-glucan levels, lower viability, and reduced tolerance to oxidative stress. We further demonstrate that the *vadA* null mutant shows increased production of the mycotoxin sterigmatocystin. In summary, VadA is a dual-function novel regulator that controls development and secondary metabolism, and participates in bridging differentiation and viability of newly formed conidia in *A*. *nidulans*.

## Introduction

Species of the genus *Aspergillus* are widespread in nature and have both beneficial and detrimental effects on humankind [1–3]. This genus includes plant and human pathogenic fungi, such as *Aspergillus fumigatus* and *Aspergillus flavus*, and other species that are of tremendous importance to the industrial production of enzymes, organic acids, and foods [1, 4]. *Aspergillus nidulans* is a model ascomycetous fungus for studying fungal development and secondary metabolism [5, 6]. All *Aspergillus* species produce asexual spores called conidia, which are the main reproductive propagule and the infectious particles [7]. Conidia are formed on a multicellular asexual structure called the conidiophore [8, 9]. Conidiophore formation is an elaborate process requiring differentiation of multiple cell types and precise regulation of several hundred genes [10, 11]. The mechanisms regulating asexual development (conidiation) have been well studied in *A*. *nidulans* [5, 6, 10].

In *A*. *nidulans*, the process of conidiation is regulated mainly by the three central regulatory genes, *brlA*, *abaA*, and *wetA* [10, 12, 13]. They control expression of conidiation-specific genes and determine the order of activation of downstream genes [12–15]. BrlA is a key element for initiation of conidiation and activates *abaA*, which in turn activates *wetA* [16–19]. WetA regulates spore-specific genes’ expression and plays a crucial role in the synthesis of spore wall components during the late phase of conidiation [20, 21]. Our previous genetic studies identified the *velvet* regulator VosA that plays a role in negative feedback regulation of *brlA* during the late phase of conidiation and in conidia [22]. In addition, VosA and VelB, together with WetA, regulate conidial maturation and trehalose biosynthesis [22–24].

The *velvet* regulators, including VosA, VeA, VelB, and VelC, are highly conserved in filamentous and dimorphic fungi and play a pivotal role in fungal biology [22, 25, 26]. In *Aspergillus* species, the *velvet* proteins coordinate fungal growth, development, pigmentation, and primary/secondary metabolism [22–24, 27–33]. These regulators form various homo- or hetero-complexes, such as VelB-VeA-LaeA, VosA-VelB, VosA-VelC, and VelB-VelB, controlling asexual sporogenesis, sexual fruiting, sterigmatocystin (ST) production, and spore maturation [23, 24, 27, 28]. Among these complexes, the VosA-VelB complex functions as a key functional unit controlling spore maturation, trehalose biosynthesis, and conidial germination [23, 24]. Recent studies have demonstrated that the *velvet* proteins are fungus-specific transcription factors that recognize the specific *cis*-acting responsive elements present in the promoters of direct target genes [34, 35]. The VosA-VelB heterodimer represses developmental regulatory genes but activates genes associated with spore maturation and trehalose biosynthesis in conidia [35]. Several predicted target genes that are directly regulated by the VosA-VelB heterodimer have been proposed [35]. Importantly, the VosA-VelB complex directly represses β (1,3)-glucan biosynthesis in asexual and sexual spores [36].

In this study, we characterize one activatory target gene, called *vadA* (VosA/VelB-Activated Developmental gene; AN5709). Levels of *vadA* mRNA in conidia are reduced by the deletion of *vosA* or *velB*. The *vadA* gene appears to be specifically expressed during the late phase of conidiation and in conidia. The deletion of *vadA* results in the enhanced production of sexual fruiting bodies (cleistothecia) and altered expression of *brlA*, *vosA*, and *velB*. The Δ*vadA* conidia exhibit reduced trehalose levels, lowered viability, and increased sensitivity to oxidative stress. In addition, the deletion of *vadA* causes increased β(1,3)-glucan accumulation in asexual spores. Genetic studies demonstrated that VadA is required for proper regulation of *brlA*, *fksA* (1,3-beta-glucan synthase), *rodA* (hydrophobins), *tpsA* (trehalose-6-phosphate synthase), and *orlA* (trehalose 6-phosphate phosphatase) in asexual spores. Overexpression (OE) of *vadA* causes enhanced formation of asexual spores. We further demonstrated that Δ*vadA* strains showed increased ST production. Overall, these results suggest that VadA is a multifunctional sporogenesis regulator controlled by the VosA-VelB complex in *A*. *nidulans*.

## Materials and methods

### Strains, media, and culture conditions

Fungal strains used in this study are listed in Table 1. All media used in this study have been previously described [28, 37]. Briefly, liquid or solid minimal media with 1% glucose (MMG) were used for general purposes and sexual medium (SM) was used for enhancing sexual development. To examine the effects of overexpression of *vadA* under the *alcA* promoter [38, 39], tested strains were inoculated on MMG or MM with 100 mM threonine as the sole carbon source (MMT). All strains were grown on solid or liquid media with appropriate supplements at 37°C. To check the number of conidia and cleistothecia, wild-type (WT), mutants, and complemented strains were point inoculated and cultured on solid MM or SM for four or seven days at 37°C. *Escherichia coli* DH5α cells were grown in Luria–Bertani medium with ampicillin (100 μg/mL) for plasmid amplification.

**Table 1:** *Aspergillus* strains used in this study.

Strain name	Relevant genotype	References
FGSC4	*A*. *nidulans* wild type, *veA*^*+*^	FGSC^a^
RJMP1.59	*pyrG89*; *pyroA4*; *veA*^+^	[40]
TNJ36	*pyrG89; AfupyrG*^+^; *pyroA4*; *veA*^+^	[41]
THS15.1	*pyrG89*; *pyroA4*; Δ*vosA*::*AfupyrG*^+^; *veA*^+^	[23]
THS16.1	*pyrG89*; *pyroA4*; Δ*velB*::*AfupyrG*^+^; *veA*^+^	[23]
THS30.1	*pyrG89*; *AfupyrG*^+^; *veA*^+^	[36]
THS33.1~3	*pyrG89*; *pyroA4*; Δ*vadA*::*AfupyrG*^*+*^; *veA*^*+*^	This study
THS34.1	*pyrG89*; *pyroA*::*vadA(p)*::*vadA*::FLAG_3x_::*pyroA*^*b*^; Δ*vadA*::*AfupyrG*^*+*^; *veA*^*+*^	This study
THS40.1	*pyrG89; AfupyrG*^*+*^; *pyroA*::*alc(p)*::*vadA*::FLAG::*pyroA*^*b*^; *veA*^*+*^	This study

^a^ Fungal Genetic Stock Center

^b^ The 3/4 *pyroA* marker causes targeted integration at the *pyroA* locus.

For Northern blot analysis, samples were collected as previously described [28]. Briefly, for conidia, the conidia of WT and mutant strains were spread onto solid media and incubated at 37°C. After two days of culture, the conidia were filtered, collected, and stored at −80°C. For hyphal samples, conidia of WT and mutant strains were inoculated into 200 mL liquid MM in 1 L flasks and incubated at 37°C. Samples of the submerged cultures were collected at designated time points and stored at -80°C. For developmental induction, the conidia of WT and mutant strains were inoculated into liquid MM and incubated for 18 h. Mycelia were filtered, washed, and spread in a monolayer on solid MM, and the plates were air exposed for asexual developmental induction, or sealed of air and blocked from light for sexual developmental induction.

### Construction of the *vadA* mutants

The oligonucleotides used in this study are listed in Table 2. The *vadA* deletion (Δ*vadA*) mutant strain was generated by double-joint PCR (DJ-PCR) as previously described [42]. The flanking regions of the *vadA* gene were amplified using the primer pairs OHS859/OHS861 and OHS860/OHS862 from *A*. *nidulans* FGSC4 genomic DNA as a template. The *A*. *fumigatus pyrG* marker was amplified from *A*. *fumigatus* AF293 genomic DNA with the primer pair OJH84/OJH85. The *vadA* deletion cassette was amplified with primer pair OHS863/OHS864 and was introduced into a RJMP1.59 [40] protoplasts generated by the Vinoflow FCE lysing enzyme (Novozymes) [43]. To complement Δ*vadA*, the WT *vadA* gene region, including its predicted promoter, was amplified with the primer pair OHS888/OHS889, digested with *Eco*RI and *Not*I, and cloned into pHS13 [23]. The resulting plasmid pHSN85 was then introduced into the recipient Δ*vadA* strain THS33.1 to give rise to THS34.1. To generate the *alcA*(p)::*vadA* fusion construct, the *vadA* ORF derived from genomic DNA was amplified using the primer pair OHS887/OHS889. The PCR product was then double digested with *Eco*RI and *Not*I and cloned into pHS82 [23]. The resulting plasmid pHS82 was then introduced into RJMP1.59. The *vadA*-overexpressing strains among the transformants were screened by Northern blot analysis using a *vadA* ORF probe followed by PCR confirmation.

**Table 2:** Oligonucleotides used in this study.

Name	Sequence (5′ → 3′)^a^	Purpose
**OHS753**	ATGTCTCCCAGACCACCAAGTATC	5′ *vadA* probe
**OHS754**	GGCTTGAGCTTGGATATGAACCG	3′ *vadA* probe
**OHS859**	GTGGTTGCTAGTCCGCAGAGAG	5′ flanking region of *vadA*
**OHS860**	GCTGGGTCAAACAAGCCAGTGC	3′ flanking region of *vadA*
**OHS861**	*GGCTTTGGCCTGTATCATGACTTCA* GGAGGCCGTAGTAGAGTGAAAGATG	5′ *vadA* with *AfupyrG* tail
**OHS862**	*TTTGGTGACGACAATACCTCCCGAC* CGACGAGCTGTATGCCTTCATG	3′ *vadA* with *AfupyrG* tail
**OHS863**	CTGGGTGGAGAGGCTAACTGC	5′ nested of *vadA*
**OHS864**	ACTCCAGCGCATTATCCAACTCAG	3′ nested of *vadA*
**OHS887**	ATAT**GAATTC**ATGTCTCCCAGACCACCAAGTATC	5′ *vadA* with *Eco*RI
**OHS888**	ATAT**GAATTC**TGCTCCTGGTAAGAAATGCTCG	5′ *vadA* with pro and *Eco*RI
**OHS889**	ATAT**GCGGCCGC**CAAGTTAGTGATGTACTCTTTCATATCC	3′ *vadA* with *Not*I
**OJH84**	GCTGAAGTCATGATACAGGCCAAA	5′ *AfupyrG* marker
**OJH85**	ATCGTCGGGAGGTATTGTCGTCAC	3′ *AfupyrG* marker
**OJA142**	CTGGCAGGTGAACAAGTC	5′ *brlA* probe
**OJA143**	AGAAGTTAACACCGTAGA	3′ *brlA* probe
**OJA150**	CAGTACGTCAATATGGAC	5′ *wetA* probe
**OJA151**	GTGAAGTTGACAAACGAC	3′ *wetA* probe
**OJA156**	ATGCTATATTCACCACCT	5′ *rodA* probe
**OJA157**	TGACCTACCAGAATATCG	3′ *rodA* probe
**OMN66**	TTTCCAGATCCTTCGCAG	5′ *vosA* probe
**OMN63**	ATAGAAACAGCCACCCAG	3′ *vosA* probe
**OMN125**	TATGCACTGGCACTCAAGCAACCG	5′ *velB* probe
**OMN126**	GTGCATGACGGTCGTATCTGGTCC	3′ *velB* probe
**OMN143**	ACTTATGCCAACGTTCTGCG	5′ *fksA* probe
**OMN144**	AAAGAGCGGGCAGCATAATG	3′ *fksA* probe
**OMN176**	CCATCACCATAAAGCGATCAG	5′ *tpsA* probe
**OMN177**	CAGTTTCGAGAAGTTAAGCGC	3′ *tpsA* probe
**OMN182**	CAGCCGCATCTCCAACTTAG	5′ *orlA* probe
**OMN183**	TGTTAGCAGCAATTCATGGCG	3′ *orlA* probe

^**a**^ Tail sequences are shown in italics. Restriction enzyme sites are in bold.

### Nucleic acid isolation and manipulation

Total RNA isolation and Northern blot analyses were performed as previously described [42, 44]. The DNA probes for Northern blot analysis were amplified using the appropriate oligonucleotide pairs (Table 2) from the coding regions of individual genes using FGSC4 genomic DNA as a template. ^32^P-labeled probes were prepared using the Random Primer DNA Labeling Kit (Clontech) with [α-^32^P]-dCTP. Genomic DNA extractions were carried out as previously described [43].

### Spore viability test

To check spore viability, fresh conidia from two-day-old cultured WT and mutant strains were spread on solid MM and incubated at 37°C. Conidia from five- and ten-day-old cultures were then collected. About 100 conidia were inoculated onto solid MM and incubated at 37°C for 48 h in triplicate. Survival rates were calculated as the ratio of the number of viable colonies to the number of spores inoculated.

### Spore trehalose assay

The spore trehalose assay was performed as previously described [22]. Briefly, conidia from two-day-old cultured WT and mutant strains were collected, washed with ddH_2_O, resuspended in 200 μL of ddH_2_O, and incubated at 95°C for 20 min. The supernatant was collected by centrifugation, mixed with an equal volume of 0.2 M sodium citrate (pH 5.5), and incubated at 37°C for 8 h with or without (as a control) 3 mU of trehalase (Sigma, St Louis, MO, USA). The amount of glucose generated from the trehalose was assayed with a Glucose Assay Kit (Sigma, St Louis, MO, USA) in triplicate.

### Oxidative stress tolerance test

The hydrogen peroxide sensitivity of conidia was tested by spotting 10 μL of serially diluted conidia (10 to 10^5^) on solid MM with 0 and 2.5 mM of H_2_O_2_ and incubating at 37°C for 48 h.

### β(1,3)-glucan analysis

The β(1,3)-glucan assay was performed as previously described [36]. Briefly, two-day old conidia of WT and mutants were collected in ddH_2_O. Conidia suspensions (10^2^ to 10^5^) were resuspended in 25 μL of ddH_2_O and were mixed with Glucatell^®^ reagents (Associates of Cape Cod, East Falmouth, MA, USA) following the manufacturer’s instructions. After incubation, diazo reagents were added to stop the reaction. The optical density at 540 nm was determined. This test was performed in triplicate.

### Sterigmatocystin (ST) extraction and HPLC conditions

Briefly, 10^6^ conidia of each strain were inoculated into 2 mL liquid complete medium (CM) and cultured at 30°C for 7 days. Secondary metabolites were extracted by adding 2 mL of CHCl_3_, the organic (CHCl_3_) phase separated by centrifugation and transferred to new glass vials, evaporated in the fume hood, and resuspended in 1 mL HPLC-grade acetonitrile:methanol (50:50, v/v). The samples were filtered through a 0.45-μm pore filter.

High-performance liquid chromatography with diode-array detection (HPLC-DAD) analysis was performed with a Series 1100 binary pump with an auto sampler and Nova-Pak C-18 column (Agilent Technologies, Waldbronn, Germany). Ten microliters of the samples were injected in to the column. The mobile phase consisted of acetonitrile:water (60:40, v/v). The flow rate was 0.8 mL/min. The ST stock solution (Sigma, St Louis, MO, USA) was dissolved in acetonitrile:methanol (50:50, v/v). A linear calibration curve (*R*^2^ = 0.998) was constructed with a ST dilution series of 10, 1, 0.5, 0.1, and 0.005 μg/mL. ST was detected at a wavelength of 246 nm. The retention time for ST was approximately 5.6 min.

### Microscopy

The colony pictures were taken using Sony digital (DSC-F828) camera. Micrographs were taken using a Zeiss M2Bio microscope equipped with AxioCam and AxioVision digital imaging software.

### Statistical analysis

Statistical differences between WT and mutant strains were evaluated by Student’s unpaired t-test. Mean ± SD are shown. P values < 0.05 were considered to be significant.

## Results

### Expression and phylogenetic analyses of *vadA*

**Fig 1:**
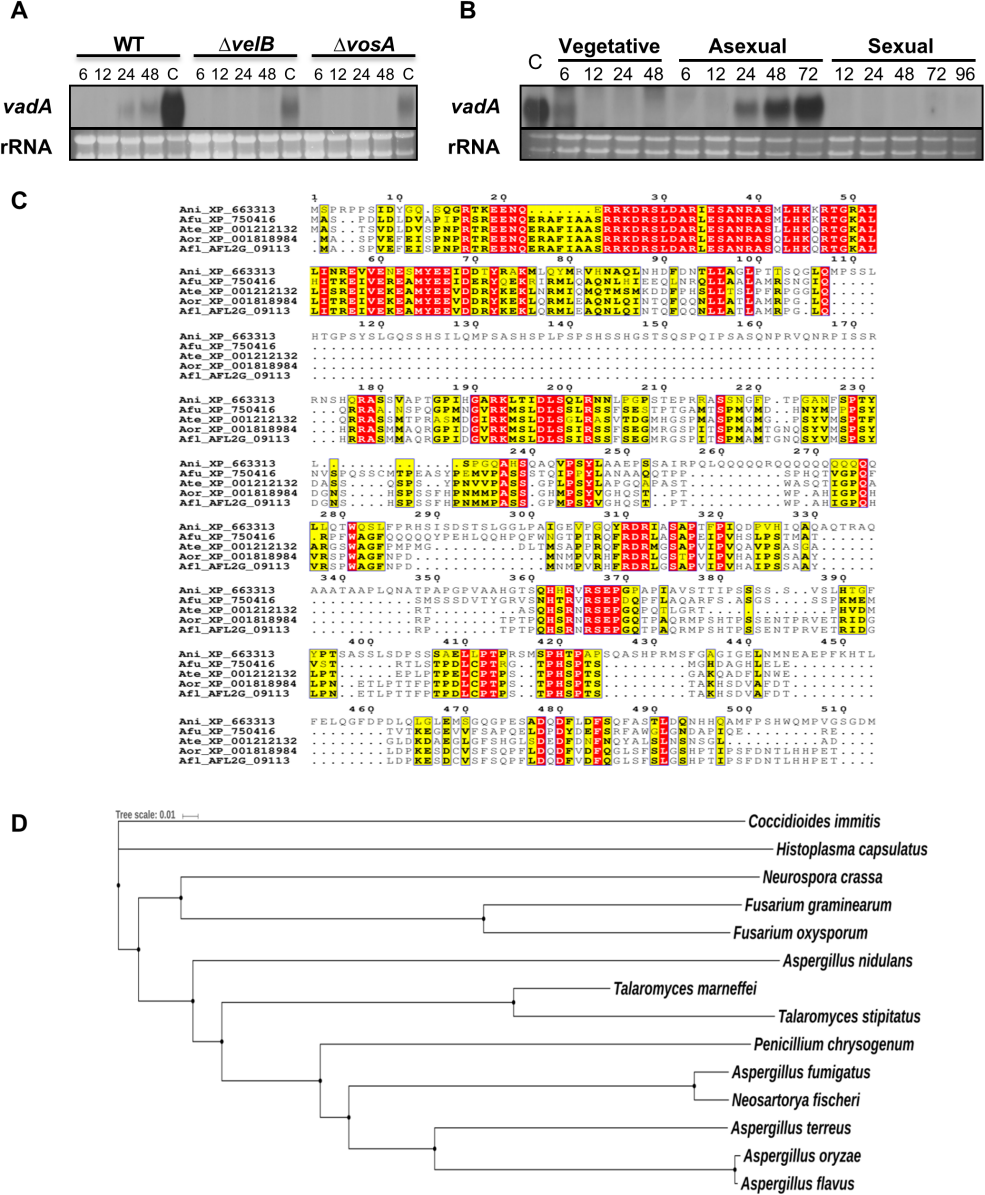
Summary of *vadA*. **(A)** Northern blot showing levels of *vadA* mRNA in WT (FGSC4), Δ*vosA* (THS15.1), and Δ*velB* (THS16.1) strains. Conidia (asexual spores) are indicated as C. The time (hours) of incubation in post asexual developmental induction is shown. Equal loading of total RNA was confirmed by ethidium bromide staining of rRNA. **(B)** Levels of *vadA* mRNA during the lifecycle of *A*. *nidulans* WT (FGSC4). Conidia (asexual spores) are indicated as C. Time (h) of incubation in liquid submerged culture and post-asexual or sexual developmental induction is shown. Equal loading of total RNA was confirmed by ethidium bromide staining of rRNA. **(C)** Alignment of *A*. *nidulans* VadA (Ani: XP_663313), *A*. *fumigatus* VadA (Afu: XP_750416), *A*. *terreus* VadA (Ate: XP_001212132), and *A*. *oryzae* VadA (Aor: XP_001818984) with VadA of *A*. *flavus* (Afl: AFL2G_09113). ClustalW2 (http://www.ebi.ac.uk/Tools/msa/clustalw2/) and ESPript (http://espript.ibcp.fr/ESPript/ESPript/ [45]) were used for the alignment and presentation. **(D)** A phylogenetic tree of VadA-like proteins identified in various fungal species including *A*. *nidulans* FGSC4 (XP_663313), *A*. *terreus* NIH2624 (XP_001212132), *A*. *oryzae* RIB40 (XP_001818984), *A*. *fumigatus* Af293 (XP_750416), *A*. *flavus* AFL3357 (AFL2G_09113), *Talaromyces marneffei* ATCC18224 (XP_002144293), *T*. *stipitatus* ATCC10500 (XP_002341254), *Penicillium chrysogenum* Wisconsin 54–1225 (XP_002562176), *Neosartorya fischeri* NRRL181 (XP_001264997), *Neurospora crassa* OR74A (XP_959987), *Coccidioides immitis* RS (XP_001244564), *Histoplasma capsulatus* G186AR (EEH08887), *Fusarium graminearum* PH1 (XP_011326731), and *F*. *oxysporum* Fo47(EWZ44948). A phylogenetic tree of putative VadA orthologues was generated by MEGA 5 software (http://www.megasoftware.net/) using the alignment data from ClustalW2. The tree results were submitted to iTOL (http://itol.embl.de/) to generate the figure.

Our previous data showed that the accumulation of *vadA* mRNA in conidia is dependent on both VosA and VelB [35]. To further verify the *vadA* expression during asexual development, we examined the levels of *vadA* mRNA in WT, Δ*vosA*, and Δ*velB* strains under asexual developmental conditions. While accumulation of *vadA* mRNA was detectable at 24 h after developmental induction and was high in the WT conidia, *vadA* mRNA was decreased in both the Δ*vosA* and Δ*velB* strains compared to WT (Fig 1A). We also examined levels of the *vadA* transcript throughout the life cycle and found that the levels of the *vadA* transcript were high during the late phase of asexual development (Fig 1B). These results suggest that *vadA* is specifically expressed during conidiogenesis, and its expression is largely dependent on both VelB and VosA. Multiple sequence alignment of *Aspergillus* spp. suggests that the predicted VadA protein contains a highly conserved domain of unknown function (DUF) at the N-terminus (Fig 1C). VadA orthologues are found in many Ascomycota (*Neurospora*, *Sordaria*, and *Marssonina*) and some Glomeromycota (*Rhizophagus*), implying that this DUF might be ancient, and has uniquely evolved in fungi, but not plants and animals (Fig 1D).

### VadA balances development

**Fig 2:**
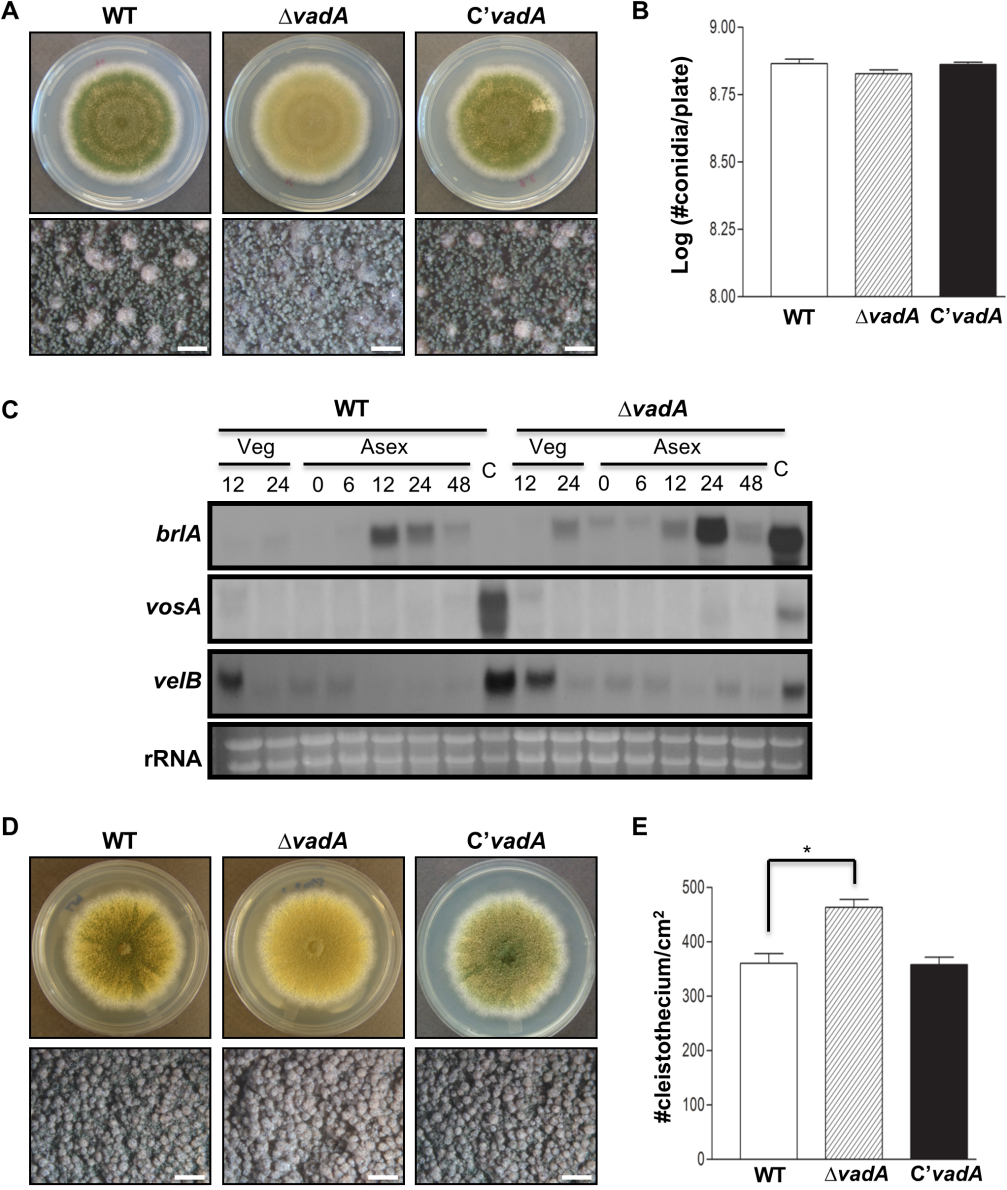
Developmental phenotypes of the Δ*vadA* mutant. **(A)** Colony photographs of WT (FGSC4), Δ*vadA* (THS33.1), and the complemented (THS34.1) strains point inoculated on solid MM and grown for four days (top and bottom panels). The bottom panel shows close-up views of the center of the plates. (bar = 0.5 mm) **(B)** Quantitative analysis of conidiospore formation of the strains shown in (A). **(C)** Northern blot for *brlA*, *vosA*, and *velB* mRNAs in WT (FGSC4) and Δ*vadA* (THS33.1) strains in vegetative growth (Veg) and post-asexual developmental induction (Asex). Numbers indicate the time (h) of incubation after induction of asexual development. Equal loading of total RNA was confirmed by ethidium bromide staining of rRNA. **(D)** Colony photographs of WT (FGSC4), Δ*vadA* (THS33.1), and the complemented (THS34.1) strains point inoculated on solid SM and grown for five days (top and bottom panels). The bottom panel shows close-up views of the center of the plates. (bar = 0.5 mm) **(E)** Quantitative analysis of cleistothecia formation of strains shown in (A) (* P < 0.05).

To study functions of *vadA*, we generated the *vadA* deletion (Δ*vadA*) mutant, complemented strains (C’), and examined their phenotypes. The Δ*vadA* mutant produced light green conidia distinct from those of WT and C’ strains (Fig 2A). The Δ*vadA* mutant produced a similar number of conidia as WT and C’ strains (Fig 2B). We then checked whether the absence of *vadA* altered the patterns of *brlA*, *vosA*, and *velB* mRNA accumulation during vegetative growth and asexual development. As shown Fig 2C, *brlA* mRNA accumulation in the Δ*vadA* mutant was detectable in vegetative growth, early asexual development (~6 h), and conidia, but was not observed in WT cells. The levels of *vosA* and *velB* mRNAs in the Δ*vadA* mutant were decreased compared to that of WT. These results indicate that VadA is required for full conidial pigmentation and proper expression of asexual developmental genes.

To test whether VadA is also associated with sexual development, WT, Δ*vadA*, and the complemented strains were inoculated on SM, and the numbers of sexual fruiting bodies were counted. As shown Fig 2D and 2E, the Δ*vadA* mutant produced significantly higher numbers of sexual fruiting bodies compared to the WT and complemented strains, suggesting that VadA is also required for proper sexual development in *A*. *nidulans*.

### VadA governs the conidial integrity

**Fig 3:**
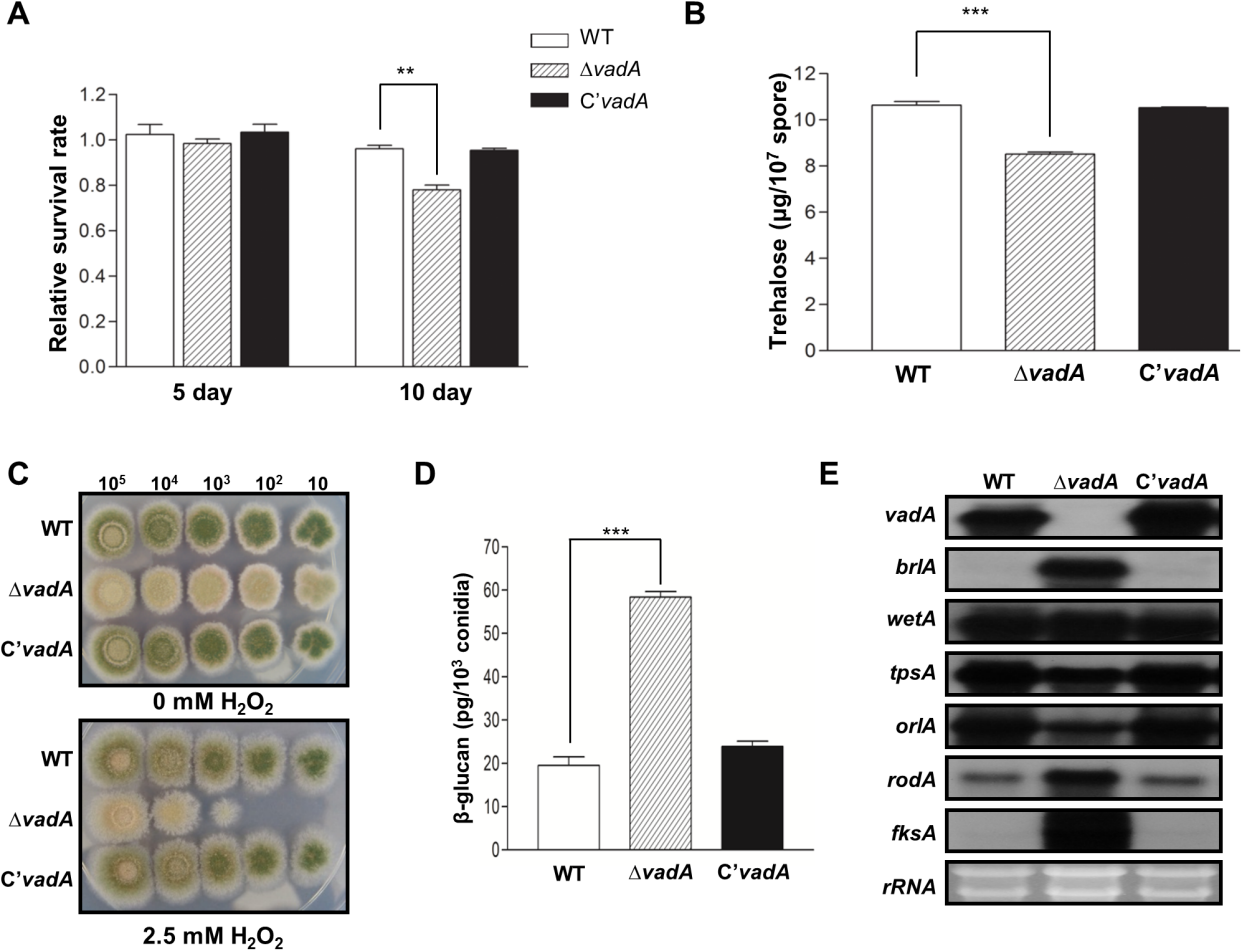
The role of *vadA* in conidia. **(A)** Viability of the conidia of WT (FGSC4), Δ*vadA* (THS33.1), and C’ (THS34.1) strains grown at 37°C for 5 and 10 days (** P < 0.01). **(B)** Amount of trehalose per 10^7^ conidia in the two-day-old conidia of WT (FGSC4), Δ*vadA* (THS33.1), and C’ (THS34.1) strains (measured in triplicate) (*** P < 0.001). **(C)** Tolerance of the conidia of WT (FGSC4), Δ*vadA* (THS33.1), and C’ (THS34.1) strains to H_2_O_2_ treatment. **(D)** Amount of β-glucan (pg) per 10^3^ conidia in the two-day-old conidia of WT (FGSC4), Δ*vadA* (THS33.1), and C’ (THS34.1) strains (measured in triplicate). **(E)** Levels of *vadA*, *brlA*, *wetA*, *tpsA*, *orlA*, *rodA*, and *fksA* transcripts in the conidia of WT (FGSC4), Δ*vadA* (THS33.1), and C’ (THS34.1) strains. Equal loading of total RNA was confirmed by ethidium bromide staining of rRNA.

As described above, *vadA* is a conidia-specific gene (Fig 1) and is required for proper expression of *brlA*, *vosA*, and *velB* in conidia (Fig 2), implying that VadA has a potential role in conidiogenesis. To test this idea, the conidial viability, trehalose content, oxidative stress tolerance, β-glucan level, and expression of genes associated with conidiogenesis were compared between WT, Δ*vadA*, and C’ strains (Fig 3). When we checked the viability of conidia of colonies grown for five and ten days, the Δ*vadA* mutant conidia had a slight loss of viability at ten days (Fig 3A). We measured trehalose in two-day conidia of WT, Δ*vadA*, and C’ strains, and showed that the trehalose content in the Δ*vadA* mutant conidia was significantly decreased compared to that in the WT and complemented strains (Fig 3B). As trehalose acts as a protectant against several stresses, we examined the conidial tolerance of the strains to oxidative stress. As shown in Fig 3C, the Δ*vadA* mutant conidia were more sensitive to oxidative stress than WT and C’ conidia. We also checked β(1,3)-glucan levels and found that its levels in the Δ*vadA* conidia were higher than that of WT and C’ conidia (Fig 3D).

To correlate phenotypic changes caused by the deletion of *vadA* with molecular events, we examined mRNA levels of *brlA*, *wetA*, *tpsA*, *orlA*, *rodA*, and *fksA* in conidia. The Δ*vadA* mutant conidia showed decreased mRNA levels of *tpsA* and *orlA*, which are associated with trehalose biosynthesis, and increased transcript levels of *brlA*, *rodA*, and *fksA*, suggesting that VadA is required for properly controlling expression of certain spore-metabolic and developmental regulatory genes in conidia (Fig 3E). Taken together, VadA is expressed during conidiogenesis and globally affects metabolism, spore wall structure, and feed-back control thereby exerting the integrity of conidia.

### The absence of *vadA* leads to elevated ST production

**Fig 4:**
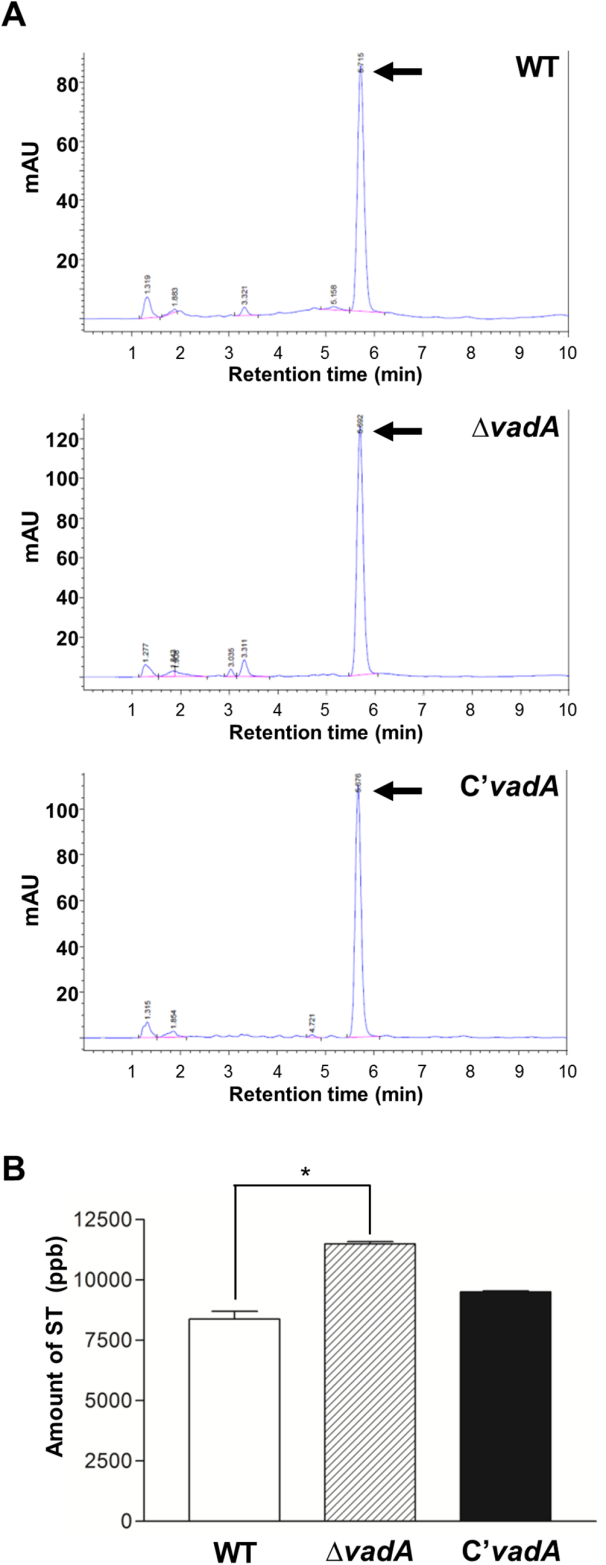
ST analysis. **(A)** Determination of ST production by WT (FGSC4) (Top), Δ*vadA* (THS33.1) (Middle), and C’ (THS34.1) (Bottom) strains. The culture supernatant of each strain was extracted with chloroform and subjected to HPLC. Arrows indicate ST. **(B)** Amount of ST produced after seven days of stationary culture (CM) of WT (FGSC4), Δ*vadA* (THS33.1) and C’ (THS34.1) strains (measured in duplicate).

We then tested whether the absence of *vadA* would affect the biosynthesis of secondary metabolites in *A*. *nidulans*. TLC image showed that all Δ*vadA* mutant strains produced increased amounts of ST compared to WT (S1 Fig). To further examine this result, we extracted ST in WT, Δ*vadA*, and C’ strains and analyzed these sample using HPLC. As shown Fig 4, the Δ*vadA* mutant produced increased amounts of ST compared to WT and C’ strains, suggesting that VadA may repress ST production in *A*. *nidulans* (Fig 4). Overall, these results imply that VadA functions controlling development, spore primary metabolism, and hyphal secondary metabolism.

### Overexpression of *vadA* leads to enhanced conidiation

**Fig 5:**
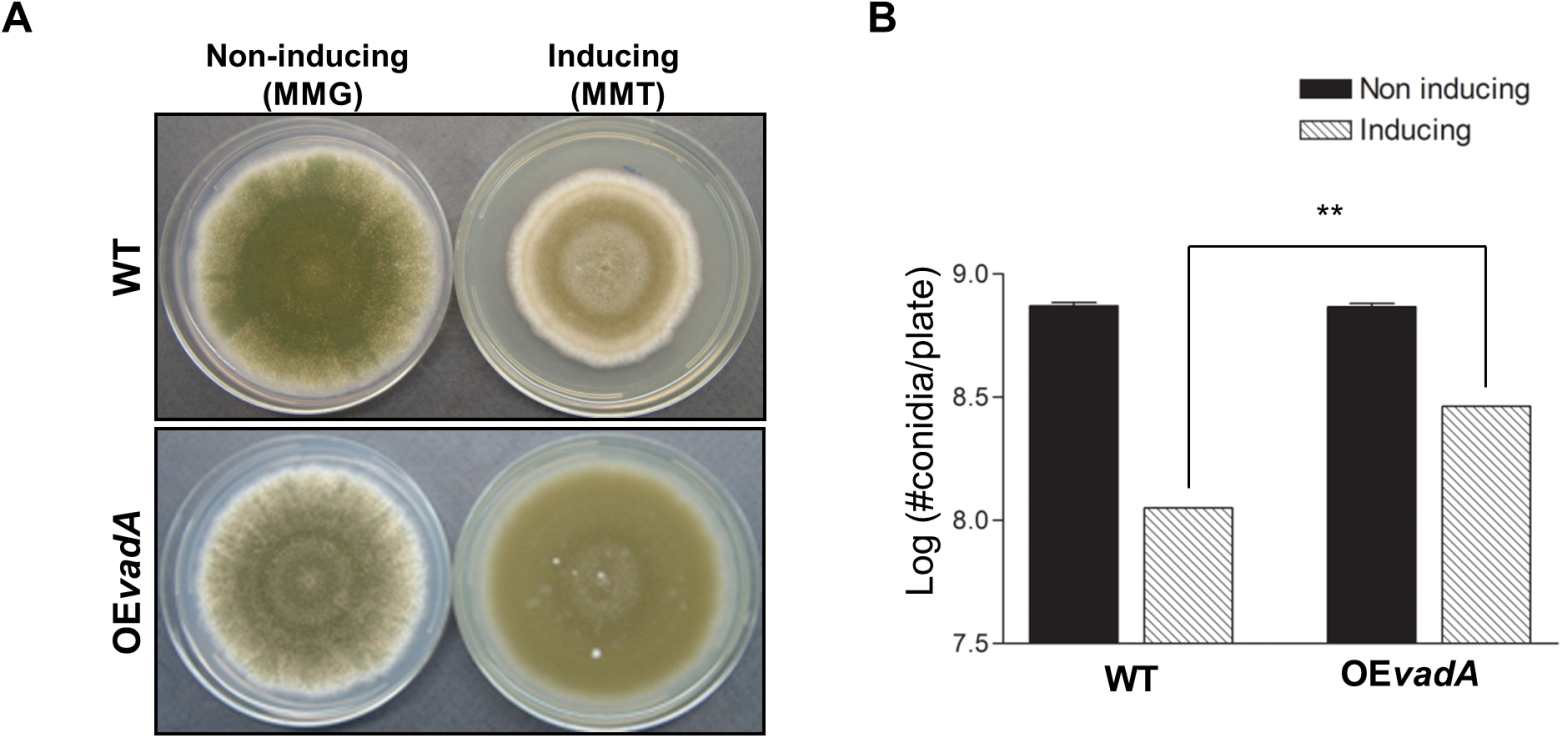
Effects of overexpression of *vadA*. (A) WT (THS30.1) and *vadA* overexpression (THS 40.1) strains were point inoculated onto solid MMG (non-inducing; left panel) or MMT (100 mM threonine, inducing; right panel) and photographed at day five. **(B)** Effects of overexpression of *vadA* on conidia formation. Quantification was done as described in the experimental procedures (** P < 0.01).

As mentioned above, the absence of *vadA* caused enhanced production of cleistothecia and altered *brlA* expression. To further test the sufficiency of VadA influencing fungal development, we constructed the *vadA* overexpression (OE) mutant and examined the developmental phenotypes upon induction. Under non-inducing conditions, the OE*vadA* mutant produced a comparable number of asexual spores compared to WT. However, when induced, overexpression of *vadA* resulted in significantly enhanced production of conidiospores (Fig 5). Collectively, these results support the idea that *vadA* is necessary for balancing asexual and sexual development, and VadA may act as an activator of asexual development.

## Discussion

The *velvet* regulators are fungal NF-κB-type transcript factors that regulate both development and metabolism [25, 35]. In particular, VosA and VelB (and their orthologues) form a hetero-complex that binds to the promoters of various developmental genes in *A*. *nidulans* and *Histoplasma capsulatum* in a sequence-specific manner [34, 35, 46]. Our previous studies demonstrated that the VosA/VelB complex regulates common targets grouped as VosA/VelB-activated developmental genes (VADs, e.g., *tpsA*) and VosA/VelB-inhibited developmental genes (VIDs, e.g., *brlA* and *fksA*), and that many VADs and VIDs are regulatory factors that subsequently control expression of downstream genes, leading to maturation of spores and completion of sporogenesis [35, 36]. The *vadA* gene is one of VADs defined in *A*. *nidulans*. In the Δ*vosA* or Δ*velB* mutant conidia, the levels of *vadA* transcript are radically decreased compare to WT conidia (Fig 1A). Our previous chromatin immunoprecipitation followed by microarray (ChIP-chip) analysis showed that the promoter region of *vadA* was enriched with VosA [35]. Based on the result of MEME (Multiple Em for Motif Elicitation) analysis, we proposed a predicted VosA-VelB binding site in the *vadA* promoter region (-245 CTACCCCAGGC -234). These results imply that the expression of *vadA* in conidia might be directly activated by VosA and VelB.

VadA is a hypothetical protein that contains a highly conserved DUF at the N-terminus (Fig 1C). The ePESTfind (http://emboss.bioinformatics.nl/cgi-bin/emboss/epestfind) program predicted that VadA has a putative PEST sequence for rapid degradation at the C-terminus (397-HTGFYPTSASSLSDPSSSAELLPTPR-422). In addition, VadA contains a nuclear localization signal (NLS)-pat4 (51-HKKR-54) and might be localized in the nucleus (56.5%), as predicted by PSORT II (http://psort.hgc.jp/form2.html). VadA is required for proper regulation of several developmental genes such as *brlA*, *fkaA*, and *rodA* in conidia. Taken together, we propose that VadA is a novel regulator involved in transcriptional control of spore-specific and metabolic genes during the lifecycle. Further studies defining the function and the molecular mechanisms for the role of VadA in sporogenesis are needed.

VadA is a conserved in many fungi including *Aspergillus* species, other Ascomycota (*Neurospora*, *Sordaria*, *and Fusarium)*, and some Glomeromycota (*Rhizophagus*). However, orthologues of VadA were not found in *Candida albicans* or *Saccharomyces cerevisiae*. To further test the role of the VadA homologues in other *Aspergillus* species, we examined the expression of *vadA* mRNAs in two major pathogens: *A*. *fumigatus* and *A*. *flavus*. Our preliminary data showed that transcript levels of the *vadA* in *A*. *fumigatus* and *A*. *flavus* are high in conidia and during the late phase of conidiation (data not shown). Moreover, in *A*. *fumigatus*, the *vadA* deletion mutant, similar to the Δ*vadA* mutant in *A*. *nidulans*, produces light green conidia that differ from WT. Taken together, these results imply that VadA is a spore-specific regulator, which plays a crucial role in sporogenesis in *Aspergillus* spp.

Although the mRNA of *vadA* is primarily detectable in conidia, VadA is also required for balanced progression of asexual and sexual development (Figs 2 and 5). The Δ*vadA* mutant exhibited increased production of sexual fruiting bodies, and overexpression of *vadA* caused elevated formation of conidiospores, suggesting that VadA may act as an activator of asexual development. We then examined the phenotypes of the *vadA* overexpression (OE) mutant strain in liquid submerged culture and found that the Δ*vadA* mutant cannot produce conidia or activate *brlA* expression, implying that VadA indirectly activates asexual development in *A*. *nidulans*.

**Fig 6:**
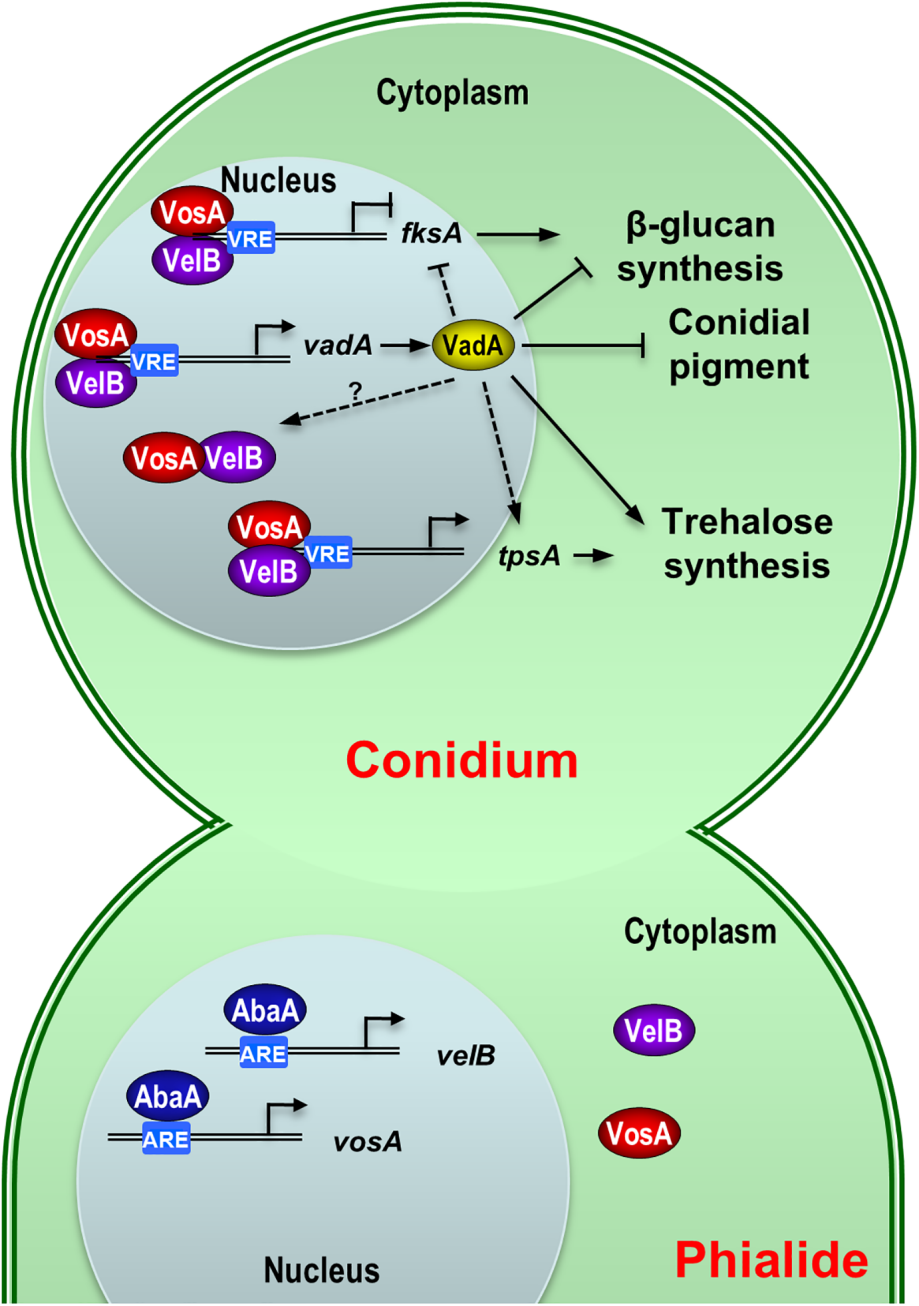
Model for VadA-mediated regulation of conidiogenesis in *A*. *nidulans*. A proposed model for the VadA-mediated conidiogenesis is presented (see Discussion).

Taken together, we present a working model for the VadA-mediated regulation of sporogenesis in *A*. *nidulans* (Fig 6). In phialides, *vosA* and *velB* are activated by AbaA [23], and the two proteins form a hetero-complex then localize in in the conidial nucleus. This VosA-VelB heterodimer binds directly to the VosA-Responsive Element (VRE) present in the promoter region of *vadA* and activates expression of *vadA*. VadA is then involved in the downregulation of *brlA*, *fksA*, and *rodA* and the proper expression of *tpsA* and *orlA* in conidia, thereby exerting the integrity and vaiblity of conidia. The molecular mechanism of *vadA*-mediated sporogenesis, as well as the genetic position of VadA, will help to understand the regulatory networks governing sporogenesis in association with VosA/VelB.

## Supporting information

S1 Fig Thin-layer chromatogram (TLC) of ST levels in WT and Δ*vadA* strains. WT (FGSC4) and Δ*vadA* (THS33.1~3) strains were stationary cultured in liquid completed medium at 30°C for 7 days. ST was extracted as described and subjected to TLC. ST standard was loaded as a positive control. Red arrow indicates ST.
(TIF)
