# Supplementary figures and images for: The role of VosA/VelB-activated developmental gene vadA in Aspergillus nidulans
Source: PLoS One. 2017 May 8;12(5):e0177099. doi: 10.1371/journal.pone.0177099 (PMC5421774; doi:10.1371/journal.pone.0177099)

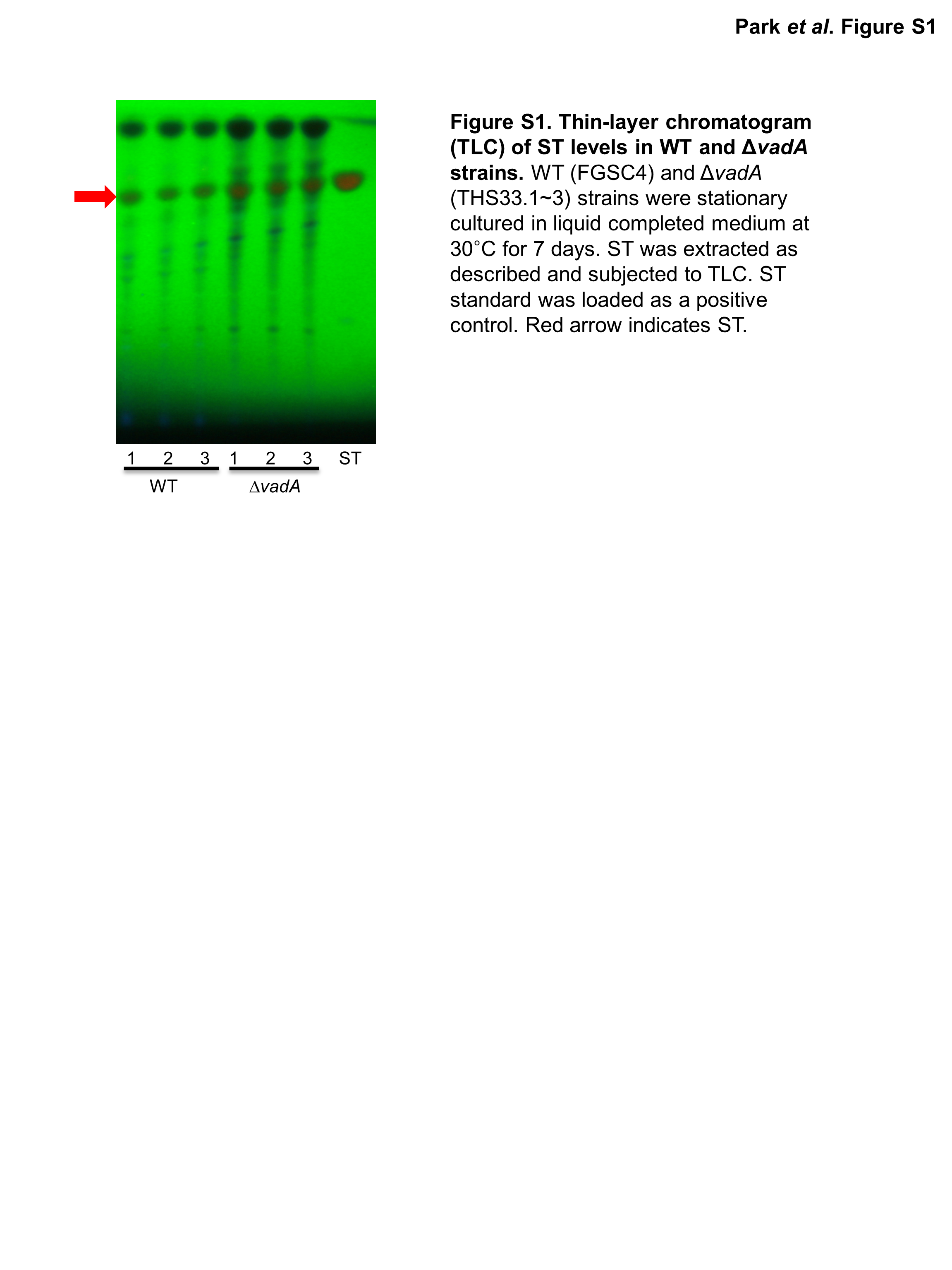

Supplement: S1 Fig — WT (FGSC4) and ΔvadA (THS33.1~3) strains were stationary cultured in liquid completed medium at 30°C for 7 days. ST was extracted as described and subjected to TLC. ST standard was loaded as a positive control. Red arrow indicates ST. (TIF) [file pone.0177099.s001.TIF]
